# Diagnostic overshadowing: An evolutionary concept analysis on the misattribution of physical symptoms to pre‐existing psychological illnesses

**DOI:** 10.1111/inm.13034

**Published:** 2022-06-19

**Authors:** Ann Hallyburton

**Affiliations:** ^1^ Hunter Library Western Carolina University Cullowhee North Carolina USA

**Keywords:** diagnostic errors, mental illness, nurse–patient relations, review [publication type]

## Abstract

This evolutionary concept analysis explores the meaning of diagnostic overshadowing within the context of physical health care for individuals with mental illness. Diagnostic overshadowing, the misattribution of symptoms of one illness to an already diagnosed comorbidity, leads to compromised patient care and likely contributes to increased mortality experienced by individuals with mental illness. A systematic literature search led to identification of a sample of 25 publications on the topic. Following Rodgers' Evolutionary Concept Analysis methodology, this literature sample yielded unifying definitions, shared themes, factors contributing to the action of diagnostic overshadowing, outcomes caused by this type of misdiagnosis, and possible interventions available to nurses and other healthcare providers. Understanding of the concept diagnostic overshadowing may help prevent its occurrence and its adverse results.

## INTRODUCTION

### Background

The Institute for Health Metrics and Evaluation (IHME) indicates 13% of the world's population has a mental illness (2019). This large population bears heavy physical as well as psychological burdens. Individuals with mental illnesses die an average of 10 years sooner than their neurotypical peers, with more than 67% of deaths attributed to heart disease and cancer (Walker *et al*. 2015). Depending upon mental illness and geographic location, decreases in average life span can exceed 20 years (Laursen *et al*. 2013; Stefancic *et al*. 2021). Such disparities extend across psychiatric diagnoses and are not limited to specific categories or levels of illness (Lawrence *et al*. 2010).

Diagnostic overshadowing presents one possible factor behind this inequity (Happell *et al*. 2016; Ho *et al*. 2021). The *Encyclopedia of Autism Spectrum Disorders* defines the term ‘diagnostic overshadowing’ as a ‘negative bias impacting a clinician's judgment regarding co‐occurring disorders in individuals who have intellectual disabilities or other mental illness’ (Kanne 2013). While not commonly listed with cognitive and clinical biases (Centre for Evidence‐Based Medicine 2021; Croskerry 2015, 2019), recognition continues to grow for this phenomenon as a type of bias within health care (Becker‐Haimes *et al*. 2021; Hinde *et al*. 2021; Jamieson & Mason 2019). Knowledge of diagnostic overshadowing is particularly vital for nurses as they are not only hands‐on care providers but lead advocates for patient care (Chuttoo & Chuttoo 2019; Martínez‐Martínez *et al*. 2021).

Concept analysis provides a useful tool for understanding the causes, meanings, and consequences of diagnostic overshadowing. Pioneered within nursing, concept analysis applies an ‘approach by which concepts that are of interest to a discipline are examined in order to clarify their characteristics or attributes’ (Cronin *et al*. 2010, p. 62). This paper offers an evolutionary concept analysis of diagnostic overshadowing applied within the context of physical illness misattributed to mental illness.

Beth Rodgers, formulator of the evolutionary concept analysis process used in this paper, discourages repeated analyses of the same concept (2018). Supporting the distinctive nature of this review, extensive searching of English language literature yielded no other published concept analysis on the topic. While the phenomenon of diagnostic overshadowing has been the subject of a meta‐analysis (White *et al*. 1995) and a qualitative systematic review (Molloy *et al*. 2021a), neither provides the in‐depth exploration of term meanings and implications afforded through concept analysis methodology.

## METHODS

### Framework

Rodgers’ (1989, 2000) evolutionary view of concept analysis encourages inquiry into how disciplines or cultures employ a concept over time. Rodgers' technique, built on the work of Wilson (1963) and Walker and Avant (1988), involves the conduct of primary activities that may take place simultaneously or out of sequence. These activities incorporate: identification of a concept, related terms, and appropriate settings and populations for exploration; data collection and analysis; exemplar presentation; and interpretation of findings, including what those findings might mean for future development of the idea (Rodgers 2000). To ensure adherence to current application of this methodology, a recent evolutionary analysis co‐authored by Rodgers (Pinto *et al*. 2017) has been consulted in concert with the original guidance (Rodgers 1989, 2000).

### Literature retrieval and analysis

#### Resources searched

Concurrent searches of 11 databases were conducted using the EBSCOhost interface: *Academic Search, Child Development & Adolescent Studies*, *CINAHL*, *Communication & Mass Media, Education Source, ERIC, MEDLINE, Military & Government Collection, APA PsycInfo, SocINDEX*, and *SPORTDiscus*. The PUBMED interface to *MEDLINE* was searched separately for in‐process materials not yet appearing in EBSCOhost's *MEDLINE*. *Google Scholar* was also searched. Searching resources from multiple areas of knowledge enables greater understanding of the concept's usage across professions (Harari *et al*. 2020). This broad view is useful, particularly due to the diverse settings in which nurses work and the varied disciplines that impact individuals affected by mental and physical illnesses.

#### Date range

A starting publication date of 2007 was selected in setting a timeline for articles included in the formal analysis. This date follows the late 2006 release of a report by the United Kingdom's Disability Rights Commission. This report (Disability Rights Commission 2006) is noted within a Joanna Briggs Institute (JBI) research protocol (Molloy *et al*. 2021b) and its resulting qualitative systematic review (Molloy *et al*. 2021a) focused on diagnostic overshadowing in a mental health context. This Disability Rights Commission report bridges usages of the term and states, ‘both people with learning disabilities and people with mental health problems experience ‘diagnostic overshadowing’, that is reports of physical ill health being viewed as part of the mental health problem or learning disability – and so not investigated or treated’ (2006, p. 6). As concept analysis methodology focuses on a concept's usage over time (Rodgers 2000), this analysis examines development of the concept within the Disability Rights Commission report and in materials published thereafter. Older publications are referenced to provide valuable historical perspective.

#### Additional criteria

To better focus the analysis, criteria in addition to publication date restrictions were set for inclusion of materials to be analysed. As the author's primary reading language is English, only English language publications were considered. Selected publications must also use the exact phrase ‘diagnostic overshadowing’ and provide a distinct definition for the term. Departing from the Disability Rights Commission's (2006) inclusion of both intellectual disabilities and mental health illnesses and in alignment with the more recent JBI research protocol (Molloy *et al*. 2021b), this analysis focuses on diagnostic overshadowing in this context as the mistaken diagnosis of physical ailments as emanating from pre‐existing psychological illness. The JBI protocol states misdiagnosis of symptoms of mental illness in individuals with intellectual disabilities as arising from their intellectual disabilities is a ‘different phenomenon’ (Molloy *et al*. 2021b, p. 1364) from misdiagnosis of symptoms of physical illness as emerging from psychiatric disorder. This exclusion is not intended to diminish diagnostic error involving intellectual disability and mental illness as a valid type of diagnostic overshadowing. Rather, application of this exclusion criteria acknowledges misdiagnosis of physical illness as psychiatric in cause constitutes a distinctive manifestation of the phenomenon.

#### Searching

Literature searches for the phrase ‘diagnostic overshadowing’ were primarily conducted in late September 2021 with follow up searches occurring in November and December 2021 and early January 2022. Search strategies were informed by guidance from Preferred Reporting Items for Systematic Reviews and Meta‐Analyses' (PRISMA) searching extension document (Rethlefsen *et al*. 2021). Searches of the noted databases carried out in late September 2021 indicated 396 academic and professional articles, reports, and indexed book chapters might meet inclusion criteria. In‐process searching of the PUBMED‐*MEDLINE* interface did not yield additional relevant materials. In addition, limited *Google Scholar* searching (review of the first 150 results) including professional and scholarly journal articles, conference presentations, graduate student publications, technical reports, and academic book publications failed to detect relevant materials not already identified in prior searches.

**Fig. 1:**
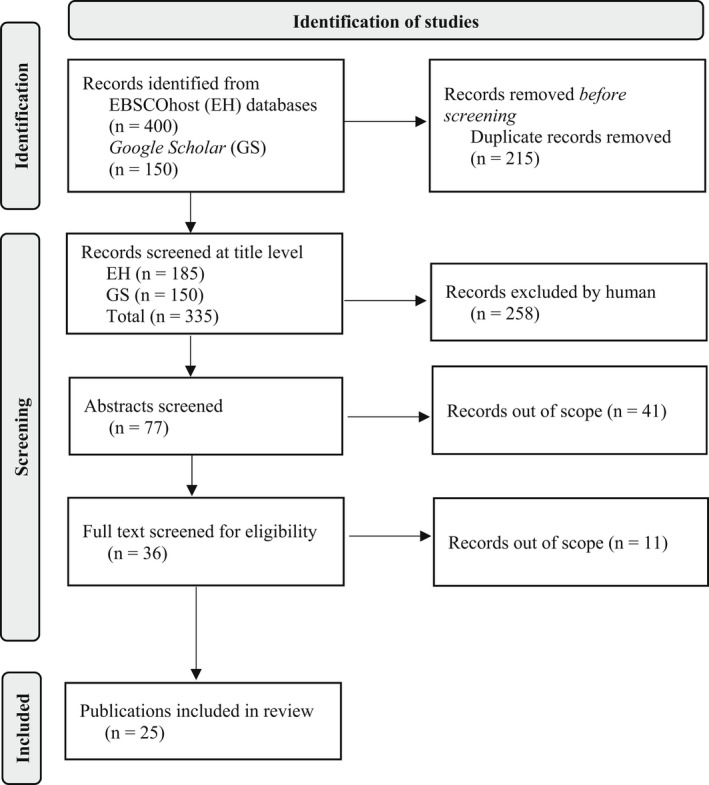
Adapted PRISMA 2020 Flow Diagram for diagnostic overshadowing searches (Page *et al*. 2021).

All results were reviewed at the title and, where warranted, abstract level (Mateen *et al*. 2013). Of these, 32 publications appeared relevant for full text review. Follow up searches yielded an additional 4 resources needing full text review. The full text of these 36 (total) publications was reviewed and 25 were found to meet inclusion criteria. References for these 25 publications were then reviewed to detect additional applicable materials. The author did not find additional relevant publications not already identified during prior searches. A scaled down reporting of results is provided (Fig. 1) and additional search information is available upon request.

Articles published within peer‐reviewed journal make up the bulk of publications selected for analysis. The 2006 Disability Rights Commission is a notable exception. The Disability Rights Commission, now subsumed within the Equality and Human Rights Commission, was a major force in human rights advocacy in the UK. As the Equity and Human Rights Commission, has received high recognition as a human rights organization from the United Nations (Equality and Human Rights Commission 2020). In addition, though the analysed article by Thornicroft *et al*. (2007) was published in the peer‐reviewed journal *International Review of Psychiatry*, it is excerpted from a 2006 book by Thornicroft.

## RESULTS

### Usage

‘Diagnostic overshadowing’ appears in English language research literature in the early 1980's to describe the misattribution of psychological issues to comorbid intellectual disabilities (Reiss *et al*. 1982). Usage morphs a quarter century later as the term is applied to the faulty attribution of physical symptoms from somatic illness to psychosomatic origins (Merrick & Merrick 2007; Thornicroft *et al*. 2007). The term has since evolved to encompass misattribution of one undiagnosed mental illness to an already diagnosed, comorbid mental illness (Becker‐Haimes *et al*. 2021; Kaufman & Baucom 2014). More recently, diagnostic overshadowing has been used to describe symptoms of undiagnosed physical illness attributed to comorbid physical ailments (Agaronnik *et al*. 2021; Barnett *et al*. 2021; Chhugani *et al*. 2021; Iezzoni 2019).

### Related concepts

Several other concepts connect with the diagnostic overshadowing. In health care, biases related to the concept of diagnostic overshadowing include anchoring, premature closure, and implicit biases (Croskerry 2019; Joint Commission 2016; Saposnik *et al*. 2016; Tsipursky 2020). Anchoring bias describes reliance upon initial impressions even after receiving additional, deviating information (Joint Commission 2016). Similarly, premature closure bias entails ceasing investigation after the formulation of an initial diagnosis (Croskerry 2019). Implicit bias involves preconceptions of race, ethnicity, gender, diagnoses, etc. and their effects on patient interaction and care (Blair *et al*. 2011; Tsipursky 2020). Such reflexive reactions may lead to missed diagnoses, ineffective therapies, and disrespectful interactions; these outcomes relate to diagnostic overshadowing as well (Joy *et al*. 2016; Stoklosa *et al*. 2017).

Terms related to diagnostic overshadowing also focus on incorrect perceptions inherent to the concept. These terms include ‘misdiagnosis’ and ‘missed diagnosis’, ‘diagnostic error’, and ‘medical error’ (Cho 2019; Jones *et al*. 2008; Molloy *et al*. 2021b). The United States National Library of Medicine's (NLM) medical subject headings' (MeSH) scope notes offer relevant descriptions for these terms as NLM's PubMed interface to MEDLINE relies upon these descriptors and PubMed is used internationally. The scope note for the heading ‘missed diagnosis’ describes the issue as ‘failure to identify or diagnose a medical condition at the time a health professional is acquainted with the symptoms’ (NLM 2020b). NLM defines ‘diagnostic errors’ as ‘incorrect or incomplete diagnoses following clinical or technical diagnostic procedures’ (2020a) and ‘medical errors’ as ‘errors or mistakes committed by health professionals, which result in harm to the patient’ (2018).

### Literature sample

While most publications describe empirical research, the sample does include editorials and theoretical pieces in which the concept is thoroughly discussed. Empirical studies primarily draw perspectives from individuals with mental illness and their healthcare providers. These providers include psychiatric care nurses, emergency department and general practice nurses and physicians, paramedics, and community healthcare workers. The author debated on whether to include both the research protocol published by JBI (Molloy *et al*. 2021b) and the resulting qualitative systematic review (Molloy *et al*. 2021a) or simply the final review alone. However, differing explanations of diagnostic overshadowing appear in the publications and research teams differ also, thereby making inclusion of both publications advisable. The 25 publications comprising this analysis emanate from Australia, Belgium, Canada, France, Norway, Portugal, the United Kingdom, and the United States (Table 1).

**Table 1:** Research context, definitions, and themes

Author(s) (Date)	Context	Diagnostic Overshadowing Definition	Theme(s)
Disability Rights Commission (2006)	Qualitative research; UK; focus groups made up of individuals with intellectual disability and/or mental illness	‘Reports of physical ill health being viewed as part of the mental health problem or learning disability – and so not investigated or treated’ (Disability Rights Commission 2006, p. 6)	Individuals with MI feel mistrust of health services provided, feel labelled by health practitioners as ‘difficult’ and unable to remove the label
Clarke *et al*. (2007)	Qualitative research; Canada; focus groups made up of 27 emergency department clients with mental illness, 7 client family members, 5 community stakeholders	‘Tendency to triage as ‘mental health’ based on history rather than on presentation’ (Clarke *et al*. 2007, p. 127)	Wait time; staff attitudes making patients with mental illness feel unimportant, shamed, guilted, stigmatized; diagnostic overshadowing; lack of treatment options; family needs
Thornicroft *et al*. (2007)	Qualitative research; UK; case studies of individuals with MI participating in community psychiatry; publication excerpted from book *Shunned: Discrimination Against People with Mental Illness* (Thornicroft 2006); published in internationally relevant, peer‐reviewed psychiatric journal	‘Misattribution of physical illness signs and symptoms to concurrent mental illness, leading to underdiagnosis and mistreatment of the physical conditions’ (Thornicroft *et al*. 2007, p. 113)	Stigma, underestimating, underlying threat of treatment through coercion/unwanted treatment, feeling punished by healthcare staff
Jones *et al*. (2008)	Opinion piece / editorial; published in internationally relevant, peer‐reviewed psychiatric journal	‘Process by which physical symptoms are misattributed to mental illness’ (Jones *et al*. 2008, p. 169)	Need more research into comorbidity to improve physical care for people with mental illness
Wood and Tracey (2009)	Qualitative research; US; case study of 220 doctoral students in clinical and counselling psychology	‘Presence of one diagnosis interferes with the detection of other diagnoses’ (Wood & Tracey 2009, p. 218)	Practitioners reduce diagnostic overshadowing through training, feedback; should be vigilant, self‐critical
Nash (2013)	Opinion piece / best practices; UK; author presents views of mental illness charity activists and organizers on care recommendations and lessons learned for nursing audience	‘Symptoms of physical illness are attributed to the service user's mental illness’ (Nash 2013, p. 22)	Stigma, underestimation, inequity, treatment delay
van Nieuwenhuizen *et al*. (2013)	Qualitative research; UK; thematic analysis of interviews of 25 emergency department clinicians	‘Misattribution of physical symptoms to pre‐existing mental illness’ (van Nieuwenhuizen *et al*. 2013, p. 255), citing Jones *et al*. 2008	Stigma; lack of knowledge; prejudice; fear of individuals with mental illness; time pressure; disagreement that diagnostic overshadowing occurs
Giddings (2013)	Opinion piece / editorial; published in official peer‐reviewed journal of Canadian Medial Association; provides examples of interactions with patients with MI in emergency department	‘Overattribution of symptoms to any underlying or long‐term condition, resulting in missed diagnoses and improper management of conditions’ (Giddings 2013, p. 1555)	Diagnostic overshadowing likely more prevalent than clinicians believe; clinicians must be ‘attuned’
Shefer *et al*. (2014)	Qualitative research; UK; interviews with 21 nurses, 18 doctors from 4 emergency departments	‘Process by which a person with a mental illness receives inadequate or delayed treatment on account of the misattribution of their physical symptoms to their mental illness’ (Shefer *et al*. 2014, p. 2)	Stigma, time, problem communication
Holm *et al*. (2014)	Qualitative research; Norway; hermeneutic analysis of interviews with 15 older adults with depression in community health centers	‘Misattribution of physical symptoms to a pre‐existing mental illness’ (Holm *et al*. 2014, p. 2), citing Nieuwenhuizen et al. (2013)	Living with stigma, not taken seriously, not knowing whether pain is physical or mental, like living in ‘war zone’
Shefer *et al*. (2015)	Qualitative research; UK; interviews with 8 doctors, 7 nurses at 4 emergency departments	‘Misattribution of physical symptoms to mental illness’ (Shefer *et al*. 2015, p. 346)	Need for liaison between emergency and psychiatry departments
Happell *et al*. (2016)	Qualitative research; Australia; focus groups of 31 consumers of mental health services	‘Service users [sic] physical symptoms are attributed to their mental illness’ (Happell *et al*. 2016, p. 2934)	Stigma; healthcare providers dismiss physical illness as part of mental illness, fail to provide care; prejudice awareness
Joy *et al*. (2016)	Opinion piece; published in official peer‐reviewed journal of American Medical Association; mental health and ethics researchers offer recommendations on MI chart labelling in psychiatric crisis centers and emergency departments	‘Psychiatric conditions overshadow their other conditions, potentially biasing the clinician's judgement about diagnosis and treatment such that the clinician may misattribute physical symptoms to mental health problems’ (Joy *et al*. 2016, p. 1539)	Stigma; implicit bias; labelling patient records harmful
Stoklosa *et al*. (2017)	Commentary; published in ethics‐focused peer‐reviewed journal of American Medical Association; responses to MI clinical vignette provided by US‐based psychiatrist, physician, and human trafficking survivor and activist	‘A well‐described clinically and ethically problematic phenomenon in which clinicians ignore patients' general health concerns because of that patient's mental illness’ (Stoklosa *et al*. 2017, p. 29)	Implicit bias; stigma; trauma‐informed care
Geiss *et al*. (2018)	Quantitative research; US; retrospective chart review of 231 admissions from psychiatric unit of rural Level 1 Trauma Center	‘Physical and/or behavioural symptoms are inappropriately accredited to mental illness’ (Geiss *et al*. 2018, p. 327)	Patients with delirium inappropriately placed in psychiatric unit; factors of age, arrhythmia, body temperature
Hext *et al*. (2018)	Clinical review/practice recommendations; published in peer‐reviewed *British Journal of* Nursing; presents guidance for UK‐based NHS Trust hospitals in working with patients with MI and physical illness	‘Tendency of professionals to overlook the signs and symptoms of a secondary condition and, instead, attributing the behaviours to the primary condition, which may be a mental health problem, learning disability or other clinical condition’ (Hext *et al*. 2018, p. 480)	Challenging behaviours; de‐escalation; legal requirements
Cho (2019)	Opinion piece/practice recommendations; author presents exemplar vignettes of patients with MI as part of work with US National Institutes of Health Clinical Center	‘Attribution of symptoms to an existing diagnosis rather than a potential comorbid condition’ (Cho 2019, p. 37)	Intersectional framework; ‘missed diagnoses and misdiagnoses’
Chuttoo and Chuttoo (2019)	Qualitative research; UK; case study of patient with MI needing physical care; prepared for primary care nursing audience	‘Symptoms being misattributed to the patient's mental health condition rather than underlying physical causes’ (Chuttoo & Chuttoo 2019, p. 78)	Stigma; nurses' role; importance of tranquil environment
Cromar‐Hayes and Seaton (2020)	Practice recommendations/review; UK‐focused paramedic recommendations on working with patients with MI needing emergency care	‘A clinician dismisses a patient's physical complaints as part of their mental illness’ (Cromar‐Hayes & Seaton 2020, p. 23)	Stigma; making most of each patient encounter; paramedic culture change needed
Perrone McIntosh (2021)	Synthesis research; internationally relevant; scoping review of emergency department‐focused literature on emergency care for patients with MI	‘Physical and/or behavioural symptoms are inaccurately correlated to mental illness’ (Perrone McIntosh 2021, p. 9)	Stigma; emergency department care constraints; perceived and experienced patient aggression; lack of knowledge, confidence on part of emergency nurses caring for patients with mental illness
Almeida *et al*. (2022)	Quantitative research; Portugal; cross‐sectional, questionnaire‐based study; 157 psychiatrists, 72 general practitioners	Not directly defined, but diagnostic overshadowing outcome explained as ‘associated with less availability and worse medical care quality’ (Almeida *et al*. 2022, p. 7) and citing (Jones *et al*. 2008)	Stigma, autonomy, coercion, diagnostic overshadowing, labelling, parental incompetence, permanence, pity, responsibility, segregation. Psychiatrists less likely to stigmatize than general practitioners.
Fontesse *et al*. (2021)	Quantitative research; conducted across Belgium, Canada, and France; survey of nurse perceptions of patients with MI; 336 nurse respondents	‘Bias of misattributing physical symptoms to mental illness’ (Fontesse *et al*. 2021, p. 155)	Stigmatization, dehumanization, burnout
Molloy *et al*. (2021b)	Synthesis research; JBI protocol for qualitative systematic review of multi‐national research on misdiagnosis of somatic illness as MI; result is Molloy, Brand, and colleagues' (2021) publication	‘A judgement bias where health care professionals mistakenly attribute clinical manifestations of physical illness (e.g. pain, tachycardia, hypertension) to manifestations associated with a pre‐existing mental illness’ (Molloy *et al*. 2021b, p. 1363)	Discrimination, inequity, stigma
Ho *et al*. (2021)	Synthesis research; integrative review following Whittemore and Knafl (2005) methodology; reviews 7 multi‐national studies, focused on audience of mental health nurses	‘Physical health complaints being interpreted as symptoms of mental illness lead to a failure to diagnosis and treatment’ (Ho *et al*. 2021, p. 8)	Need for help for carers/caregivers, providers who specialize in providing physical care to individuals with mental illness; lack of care access and coordination; advocacy
Molloy *et al*. (2021a)	Synthesis research; qualitative systematic review of 6 multi‐national studies on misdiagnosis of somatic illness as MI; result of Molloy, Munro, and colleagues' (2021) protocol	‘A complex and life‐threatening phenomenon that occurs when physical symptoms reported by mental health consumers are misattributed to mental illness by health professionals’. (Molloy *et al*. 2021a, p. 1)	Stigma, misaligned care, professionals' lack of perspective of diagnostic picture and patients' lives

### Definitions

An editorial by Jones *et al*. (2008) entitled “Diagnostic overshadowing’: Worse physical health care for people with mental illness’ provides what appears as a foundational definition for diagnostic overshadowing in mental illness and physical health misdiagnosis. This publication describes diagnostic overshadowing as the ‘process by which physical symptoms are misattributed to mental illness’ (Jones *et al*. 2008, p. 169). Multiple articles refer to the Jones *et al*. (2008) editorial when presenting their own explanations of the concept (Almeida *et al*. 2022; Cho 2019; Geiss *et al*. 2018; Giddings 2013; Ho *et al*. 2021; Molloy *et al*. 2021a; Perrone McIntosh 2021; Shefer *et al*. 2014; Stoklosa *et al*. 2017; van Nieuwenhuizen *et al*. 2013). Even included authors not citing the editorial (e.g. Holm *et al*. 2014) refer to publications directly drawing from it.

While the Disability Rights Commission report (2006) and publications by Clarke *et al*. (2007) and Thornicroft *et al*. (2007) predate, the Jones *et al*. 2008 Jones et al. editorial employs both the term and its population of interest as parts of its title. The editorial is also more quickly digestible than the longer Disability Rights Commission report and Thornicroft's (2006) book *Shunned: Discrimination Against People with Mental Illness* (2006) from which the Thornicroft *et al*. (2007) article is drawn. Of note, psychiatry researcher Thornicroft contributed to several included publications (Jones *et al*. 2008; Shefer *et al*. 2014, 2015; van Nieuwenhuizen *et al*. 2013) as well as the editorial. Whether reliance on the editorial's definition arises from ease or expertise, the description of diagnostic overshadowing it provides is succinct and most summations in the sample do not notably deviate.

### Themes

Unifying themes (Table 1) presented in the literature sample include stigmatization and underestimation of individuals with mental illness as well as delays they experience in receiving appropriate care (Cromar‐Hayes & Seaton 2020; Geiss *et al*. 2018; Happell *et al*. 2016; Ho *et al*. 2021; Nash 2013; Stoklosa *et al*. 2017). Providers' fear of violence from patients with mental illness presents another recurring theme (Fontesse *et al*. 2021; Hext *et al*. 2018; Perrone McIntosh 2021; van Nieuwenhuizen *et al*. 2013). Providers' lack of confidence in caring for patients with mental illness is a thematic element as well (Nash 2013; Perrone McIntosh 2021; Shefer *et al*. 2015; van Nieuwenhuizen *et al*. 2013). In addition, the need for debiasing interventions and culture change emerge as significant prevention messages within several publications (Almeida *et al*. 2022; Chuttoo & Chuttoo 2019; Cromar‐Hayes & Seaton 2020; Giddings 2013; Shefer *et al*. 2014, 2015; Stoklosa *et al*. 2017; Wood & Tracey 2009). Table 1 offers an overview of conditions, definitions, and thematic elements present within the sample.

### Antecedents

In concept analysis, factors that contribute to the actions a concept describes are called antecedents (Table 2). Providers' bias toward believing that difficult‐to‐diagnose physical symptoms in individuals with mental illness are psychosomatic in nature (Jones *et al*. 2008; Joy *et al*. 2016; Molloy *et al*. 2021a,b; Nash 2013; Shefer *et al*. 2014, 2015; Thornicroft *et al*. 2007) presents a primary antecedent. The misgivings of individuals with mental illness over whether their own symptoms are real (Happell *et al*. 2016; Holm *et al*. 2014) provide another contributing factor. The ‘flagging’ of health records, which may predispose care providers to associate physical symptoms with psychological origins (Almeida *et al*. 2022; Clarke *et al*. 2007; Disability Rights Commission 2006; Happell *et al*. 2016; Joy *et al*. 2016) offers one more antecedent of diagnostic overshadowing.

**Table 2:** Antecedents and consequences of diagnostic overshadowing

Antecedents	Consequences
Provider bias (Jones *et al*. 2008; Joy *et al*. 2016; Molloy *et al*. 2021a,b; Nash 2013; Shefer *et al*. 2014, 2015; Thornicroft *et al*. 2007)	Worsening condition, decreased likelihood to seek treatment, death (Jones *et al*. 2008; Joy *et al*. 2016; Molloy *et al*. 2021a,b; Nash 2013; Shefer *et al*. 2014, 2015; Thornicroft *et al*. 2007)
Patient self‐doubt (Happell *et al*. 2016; Holm *et al*. 2014)	Increased distress, worsening condition, decreased desire to seek treatment, death (Disability Rights Commission 2006; Giddings 2013; Happell *et al*. 2016; Ho *et al*. 2021; Holm *et al*. 2014; Thornicroft *et al*. 2007)
Labelling (Almeida *et al*. 2022; Clarke *et al*. 2007; Disability Rights Commission 2006; Happell *et al*. 2016; Joy *et al*. 2016)	Worsening condition, increased pre‐judgement, death (Almeida *et al*. 2022; Disability Rights Commission 2006; Happell *et al*. 2016; Joy *et al*. 2016)

### Consequences

The literature sample also highlights consequences brought about by diagnostic overshadowing (Table 2). The antecedents of provider bias, patient self‐doubt, and chart ‘flagging’ appear to yield worsening physical conditions or death (Jones *et al*. 2008; Joy *et al*. 2016; Molloy *et al*. 2021a,b; Nash 2013; Shefer *et al*. 2014, 2015; Thornicroft *et al*. 2007). Another consequence of diagnostic overshadowing is patients' decreased willingness to seek care for ailments as they fear they may not be taken seriously (Disability Rights Commission 2006; Giddings 2013; Happell *et al*. 2016; Ho *et al*. 2021; Holm *et al*. 2014; Thornicroft *et al*. 2007). ‘Flagging’ of records may also contribute to incidents of increased prejudice and misdiagnosis (Almeida *et al*. 2022; Disability Rights Commission 2006; Happell *et al*. 2016; Joy *et al*. 2016).

## DISCUSSION

Diagnostic overshadowing as a concept encompasses multiple complicated issues: need, misunderstanding, fear, prejudice, intention, and suffering. While the phrase invites images of a diagnosis obscuring others with its bulk, the stigma attached to mental illness is itself the concealing mass (Ho *et al*. 2021; Perrone McIntosh 2021; Shefer *et al*. 2014; van Nieuwenhuizen *et al*. 2013). As a term, diagnostic overshadowing relies more upon metaphor than accuracy.

Mental illnesses often receive a good deal of metaphorical overlay as well. In *Illness as Metaphor* (1978), American writer and activist Susan Sontag posits, ‘.. illness is *not* a metaphor, and that the most truthful way of regarding illness – and the healthiest way of being ill – is one most purified of, most resistant to, metaphoric thinking’ (p. 3). The illnesses obscured by the action of diagnostic overshadowing are frequently more harmful than the mental illness blamed for disguising them. By allowing perceptions of a patient's mental illness to diminish other aspects of their health, practitioners lose valuable opportunities to help these patients avoid dire outcomes.

How might nurses and other providers prevent diagnostic overshadowing from occurring? Whether an answer is engaging in self‐critical evaluation (Wood & Tracey 2009) or receiving education on trauma‐informed care (Stoklosa *et al*. 2017), collaboration between physical and mental healthcare professionals (Shefer *et al*. 2015; van Nieuwenhuizen *et al*. 2013), changes in charting practices (Joy *et al*. 2016), or, likely, a mixture of these intervention approaches, one critical initial move must be made. This first step requires acknowledging that diagnostic overshadowing exists and that its occurrence is both widespread and serious (Giddings 2013; Happell *et al*. 2016).

## CONCLUSION

As a concept, diagnostic overshadowing began as a way of naming the issue of missed diagnoses of mental illnesses in individuals having intellectual disabilities. The term then took on the meaning of physical illness misattributed to comorbid mental illness. The term now appears in descriptions of the misdiagnosis of one physical malady as being caused by a different, already diagnosed physical illness (Agaronnik *et al*. 2021; Barnett *et al*. 2021; Chhugani *et al*. 2021; Iezzoni 2019). Further developments may occur if diagnostic overshadowing achieves wider recognition within the realm of healthcare biases (Hinde *et al*. 2021; Jamieson & Mason 2019; Molloy *et al*. 2021a,b).

Additional evolution of this concept may even render it applicable to any misattribution of symptoms to a pre‐existing diagnosis. The term is likely broad enough to encompass all such meanings, and literature suggests limited usage of this interpretation is already underway (Osborne *et al*. 2017). No matter the type of illness, reducing or eliminating misdiagnosis is crucial. When practitioners manage preconceptions of patients with mental illness and actively work with them to address modifiable risk factors (smoking, diet, physical activity), mortality differences greatly diminish (Dregan *et al*. 2020). If lessening diagnostic overshadowing for all patients yields similar benefit, then sharing the terminology is worthwhile.

Nurses continue to labour as primary providers of, and primary advocates for, patient care. These roles offer sufficient challenge without the seeming obstacle of a patient's mental illness. However, by understanding and recognizing diagnostic overshadowing, nurses can better fulfil these roles as providers and advocates. In the process, this knowledge may assist them in educating colleagues and improving both the quantity and the quality of patients' lives.

## RELEVANCE TO CLINICAL PRACTICE

Nursing theory and nursing practice are inextricably linked. Concept analysis provides one such important linkage. By acquiring nuanced knowledge of a concept and its meanings, connections, implications, and future directions, nurses may more expertly handle complications and opportunities presented by the issue. In the case of diagnostic overshadowing, such understanding should lead to recognizing and preventing its occurrence, improving patient‐provider relationships, and preventing unnecessary loss of life.

## FUNDING INFORMATION

No funding was received for the commission of this research.

## Data Availability

Data sharing not applicable to this article as no datasets were generated or analysed during the current study.
